# Comparison of lumbopelvic fixation and iliosacral screw fixation for the treatment of bilateral sacral fractures

**DOI:** 10.1186/s13018-021-02768-w

**Published:** 2021-10-16

**Authors:** Katharina E. Wenning, Emre Yilmaz, Thomas A. Schildhauer, Martin F. Hoffmann

**Affiliations:** Department of General and Trauma Surgery, BG University Hospital Bergmannsheil Bochum, Buerkle de la Camp-Platz 1, 44789 Bochum, Germany

**Keywords:** Pelvic ring injury, Bilateral sacral fracture, Lumbopelvic fixation, Iliosacral screw, Retrograde medullary superior pubic ramus screw

## Abstract

**Background:**

Bilateral sacral fractures result in traumatic disruption of the posterior pelvic ring. Treatment for unstable posterior pelvic ring fractures should aim for fracture reduction and rigid fixation to facilitate early mobilization. Iliosacral screw fixation (ISF) and lumbopelvic fixation (LPF) were recommended for the treatment of these injuries. No algorithm or gold standard exists for surgery of these fractures.

**Purpose:**

The purpose of this study was to evaluate the differences between ISF and LPF in bilateral sacral fractures regarding intraoperative procedures, complications and postoperative mobilization. The secondary aim was to determine whether demographics influence surgical treatment.

**Methods:**

Over a 4-year period (2016–2019), 188 consecutive patients with pelvic ring injuries were treated at one academic level 1 trauma center and retrospectively identified. Fractures were classified according to the AO/OTA classification system. Seventy-seven patients were treated with LPF or ISF in combination with internal fixation of pubic rami fractures and could be included in this study. Comparisons were made between demographic and perioperative data. Infection, hematoma and hardware malpositioning were used as complication variables. Mobilization with unrestricted weight bearing was used as outcome variable. Follow-up was at least 6 months postoperatively.

**Results:**

Operative stabilization of bilateral posterior pelvic ring injuries was performed in 77 patients. Therefore, 29 patients (females 59%) underwent LPF whereas 48 patients (females 83%) had bilateral ISF. The ISF group was older (76 yrs.) compared to the LPF group (62 yrs.) (*p* = 0.001), but no differences regarding BMI or comorbidities were detected. Time for surgery was reduced for patients who were treated with ISF compared to lumbopelvic fixation (73 min vs. 165 min; respectively, *p* < 0.001). But this did not result in reduced fluoroscopic time or radiation exposure. Overall complication rate was not different between the groups. Patients with LPF had a greater length of stay (*p* = 0.008) but were all weight bearing as tolerated when discharged (*p* < 0.001).

**Conclusion:**

Bilateral posterior pelvic ring injuries of the sacrum can be sufficiently treated by LPF or ISF. LPF allows immediate weight bearing which may benefit younger patients and patients with an elevated risk for pneumonia or other pulmonary complications. Treatment with ISF reduces operative time, length of stay and postoperative wound infection. Elderly patients may be better suited for treatment with ISF if there is concern that the patient may not tolerate the increased operative time.

## Background

Pelvic injuries often involve the sacrum which functions as the keystone of the pelvic ring [1]. In most cases, disruption of the posterior pelvic ring is related to two different entities. In younger patients, sacral fractures occur related to high-energy trauma [2] whereas low-energy mechanisms like fall from standing height are the most common trauma mechanisms in the geriatric population [3, 4]. Historical treatment of these fractures usually consisted of non-operative treatment modalities all resulting in prolonged bed rest and insufficient reduction and/or stability. A decrease of mortality of these injuries can be achieved by surgical stabilization [5]. However, optimal treatment of sacral fractures continues to be challenging due to complex local anatomy, unique biomechanical forces and often poor bone quality especially in elderly patients [6].

Different operative stabilization techniques including open or percutaneous iliosacral screw fixation (ISF), tension band transiliac plate osteosynthesis, transiliac bars, local plate osteosynthesis and lumbopelvic fixation (LPF) were described [7–9]. When comparing these procedures, LPF seems to be biomechanically superior [10, 11] by transferring vertical loads from the ilium directly to the lumbar spine. It also prevents flexion of the pelvis in bilateral, U- or H-shaped fractures offering the possibility to salvage highly unstable pelvic ring injuries [12]. Concern exists that increased muscle mobilization and potential devitalization in case of LPF increase the risk for deep hematoma formation and infection [12, 13].

In contrast, the ISF technique introduced by Routt in the early 1990s is a quick and minimal invasive procedure [14]. It has become a commonplace technique to supplement reduction of posterior pelvic ring fractures [15]. Surgeons must be knowledgeable about individual patient anatomy to ensure safe screw placement. For this reason, some authors describe navigation techniques to increase the precision of screw placement [16]. Its disadvantages, however, are related to the necessity of anatomical or near anatomical reduction and limited biomechanical stability [11]. This instability is enhanced by additional injuries of the anterior pelvic ring which were found in the majority of patients with bilateral sacral fractures [17]. Without anterior stabilization, the hemipelvis shows paradoxical posterior sagittal plane rotation and posterior translation at the sacroiliac joint (SIJ) [18]. Therefore, additional fixation of the anterior pelvic ring was recommended [18, 19]. Retrograde medullary superior pubic ramus screws offer a minimal invasive option for stabilization of the anterior pelvic ring [20].

The relative paucity of these procedures and a widely heterogeneous patient population has resulted in few investigations regarding clinical outcome or best treatment for unstable bilateral fractures of the posterior pelvic ring [12, 21]. Therefore, the purpose of this study was to evaluate the differences between ISF and LPF in bilateral sacral fractures regarding intraoperative procedures, complications and postoperative mobility. The secondary aim was to determine whether demographics influence surgical treatment and indication.

## Methods

This study was an Institutional Review Board-approved retrospective cohort study of consecutive patients undergoing surgical treatment of bilateral sacral fractures treated with LPF or ISF in a single level I trauma center. During the study period, a total of 188 pelvic ring injuries were identified by Current Procedural Terminology (CPT) codes that had initial operative treatment for pelvic fractures from January 2016 through December 2019. Inclusion criteria were surgical treatment for unstable bilateral sacral fracture by LPF or bilateral ISF in combination with unilateral or bilateral screwing of the upper pubic ramus and age equal to or older than 18 years. Exclusion criteria were unilateral sacral fractures, ISF without superior pubic ramus screwing, metastatic disease or preexisting infection and insufficient medical record or radiographic data. No patients were phoned in specifically for this study; all data were obtained from preexisting medical records and radiographs.

Each patient had three views of the initially injured pelvis. These were an anteroposterior (AP) view with the patient supine, a pelvic inlet view, the X-ray tube angled 40º caudad and the beam centered on the umbilicus, and an outlet view, with the tube angled 60º cephalad, and the beam centered on the symphysis. Inlet and outlet views were performed for assessing rotational, translational and vertical displacement. Bilateral transforaminal sacral and transverse sacral fractures had lateral lumbosacral imaging to evaluate sacral angulation and translation. Each patient had a computed tomography (CT) scan with reconstruction of the injured pelvis that provided information on both extent of the injury and the magnitude of the displacement of the sacroiliac joint, the sacrum or the iliac wing. Furthermore, the CT defined injury to the lumbar five (L5) transverse process and/or L5/Sacral 1 (S1) facet joint injury extension. Injuries were classified according to OTA/AO [22].

**Figures 1:**
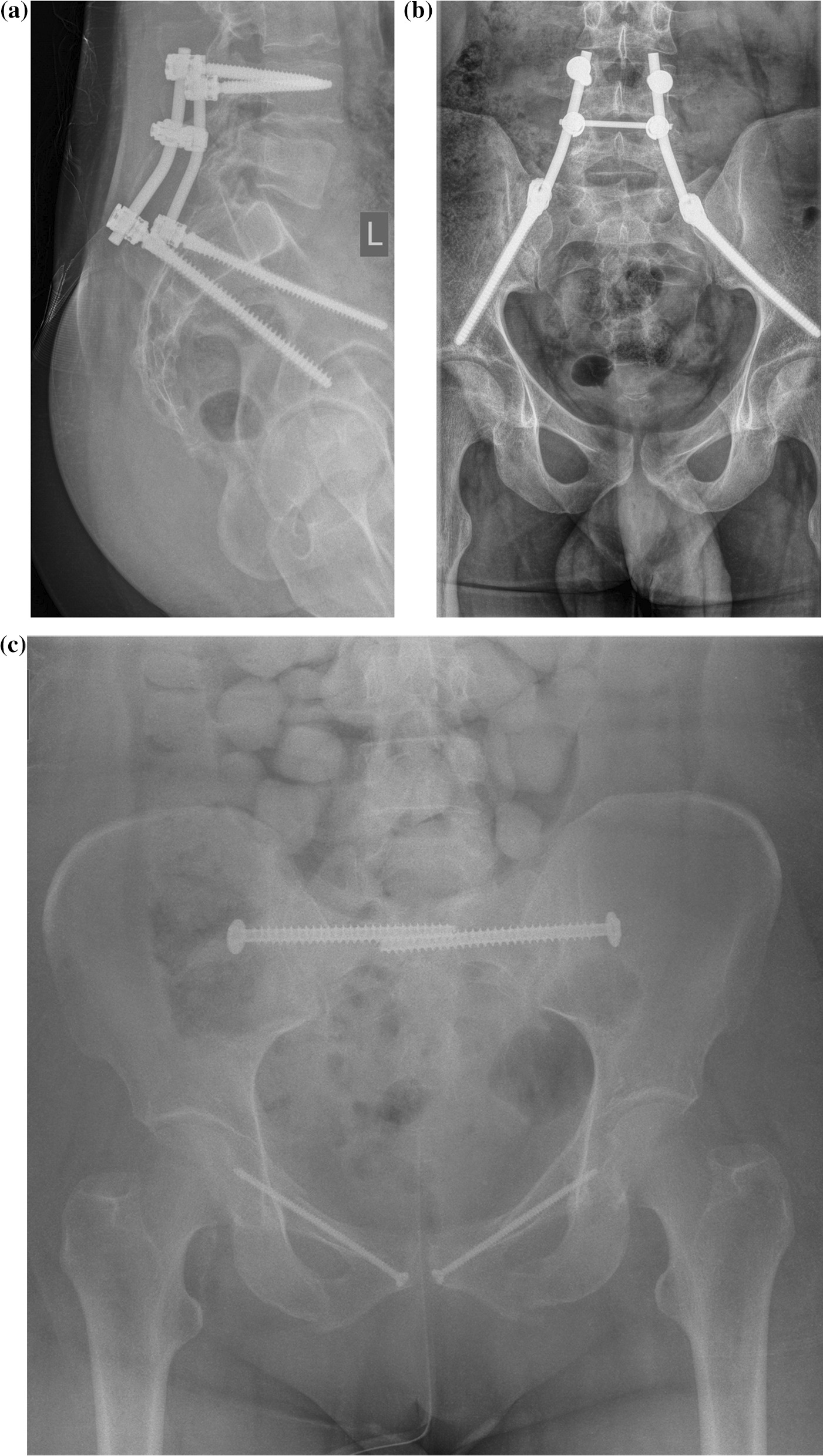
**a** and **b** Lumbopelvic implants inserted as described by Schildhauer. **c** ISF in combination with bilateral screwing of superior pubic ramus

Operative procedures were performed by four fellowship trained orthopedic trauma surgeons. Surgical indications and treatment were performed in accordance with the surgeon’s best knowledge, discretion and experience. The operative approaches to the pelvis were tailored to each patient based on the particular pattern of the injury, location of the fractures, neurologic involvement and soft tissue involvement [23]. Patients were positioned prone on a radiolucent table with appropriate eye protection and sequential compression devices for lumbopelvic fixation. Insertion of the iliosacral screws and retrograde transpubic screws was performed in a supine position. All patients underwent intraoperative fluoroscopic imaging stability examination before surgery. Lumbopelvic implants (USS, DePuySynthes, Paoli, PA) were inserted as described by Schildhauer [24] (Fig. 1a and b).

Iliosacral screws (7.3 mm, DePuySynthes, Paoli, PA) were inserted as described by Routt with additional anterior screw fixation as described by Gaensslen (Fig. 1c) [25].

Postoperatively, all patients had CT assessment of screw placement. Duplex sonography was also performed to rule out postoperative deep vein thrombosis. Thrombosis prophylaxis was carried out with a low molecular weight heparin and unfractionated heparin. Patients were mobilized based upon the constellation of injuries and operative treatment. Weight bearing was allowed following the surgeon’s discretion and recommendations. In general, non-weight bearing was continued for three months on the lower extremity for patients with SI and retrograde transpubic screw fixation. Patients with LPF were mobilized with weight bearing as tolerated. Upon beginning weight bearing, a formal therapy was instituted working on core strengthening, dynamic lumbar stabilization, range of motion, strengthening and conditioning.

Perioperative parameters such as fluoroscopy time, total radiation dose, operation time and time of hospital stay were recorded and analyzed utilizing the hospital charts. Infection, postoperative hematoma, screw malpositioning and hardware failure were utilized as complication variables. Deep infection was defined as an infection requiring operative excisional debridement and antibiotic administration.

Patients were evaluated at regular and consistent intervals of 2 weeks, 6 weeks, 12 weeks, 6 months and 1 year if possible.

### Statistical analysis

For statistical analysis Excel (Microsoft Excel for MAC, version 16.33) and SPSS version 25.0 (IBM, Chicago, IL) were utilized. To compare continuous variables (age, follow-up time, operation time, inpatient stay), T-test was performed. Fisher’s exact test was utilized to compare nominally scaled data. Significance was defined as *p* < 0.05. P-values were rounded to one decimal place.

## Results

Between January 2016 and December 2019, a total of 77 patients (20 males, 26%, and 57 females, 74%) were treated for unstable bilateral sacral fractures at one academic level 1 trauma center. LPF was performed in 29 cases (17 females, 59%) and 48 patients (40 females, 83%) received bilateral iliosacral screws in combination with (bilateral or unilateral) internal screw fixation due to pubic rami fractures. Patients of the ISF group without simultaneous fixation of the anterior pelvic ring were excluded.

### Demographic and clinical data

Looking at our study population, significantly more women underwent ISF compared to LPF (*p* = 0.02). Patients were identified with a mean age of 71 years (range 19 to 97 years). Significantly, the ISF group was older compared to the LPF group (LPF group: 62.2 ± 17.7 years, ISF group: 75.9 ± 14.0 years; *p* = 0.001). There was no significant difference between the two groups in terms of body mass index (BMI) (LPF group: 25.8 ± 5.0, ISF group: 24.0 ± 3.9; *p* = 0.10) or comorbidities (coronary heart disease, osteoporosis, diabetes mellitus).

The main commonly treated fracture patterns were 54C0 (37 patients, 48%) followed by 54C2 (26 patients, 34%) and 54C3 (14 patients, 18%) sacral spine injuries according to OTA/*AO.* The main epidemiological, clinical and radiological data regarding our patient population are summarized in Table 1.

**Table 1:** Epidemiological, clinical and radiological data of the patient population

Characteristic value	LPF group	ISF group	*P*
Number of patients (n)	29	48	
Sex (male/female) (n)	12/17	8/40	0.017
Average age (years)	62.2 ± 17.7	75.9 ± 14.0	0.001
Average HS (days)	26.6. ± 17.9	13.0 ± 7.71	0.001
BMI (kg/m2)	25.8 ± 5.0	24.0 ± 3.9	
Fracture classification			
55C0	15 (51.72%)	22 (45.83%)	
54C2	0 (0%)	26 (54.17%)	
54C3	14 (48.28%)	0 (0%)	
Average OT (min)	165.0 ± 68.0	73.0 ± 46.0	0.001
Fluoroscopy time (min)	4.88 ± 6.13	6.33 ± 4.62	
Radiation exposure (Gy*cm2)	2708.78 ± 3106.97	3136.64 ± 2066.76	
Comorbidities			
CHD (n)	17 (58.6%)	36 (75.0%)	
T2DM (n)	4 (13.8%)	5 (10.4%)	
Osteoporosis (n)	10 (34.5%)	18 (37.5%)	
Complication			
Infection (n)	4 (13.8%)	0 (0%)	0.008
Hematoma (n)	0 (0%)	1 (2.1%)	
Debridement (n)	4 (13.8)	1 (2.1%)	0.046
Screw malpositioning (n)	0 (0%)	5 (10.6%)	
Hardware failure (n)	1 (3.5%)	1 (2.1%)	
Hardware change (n)	1 (3.5%)	6 (12.8%)	
Nonunion (n)	1 (3.5%)	0 (0%)	
Bone graft (n)	1 (3.5%)	0 (0%)	
Implant removal (n)	2 (6.9%)	5 (10.6%)	
Start of weight bearing (wk)	0.83	7.0	0 001

wk = week, HS= hospital stay, OT= operation time, CHD = coronary heart disease, T2DM= type 2 diabetes mellitus

Significance of italics was defined as *P* < 0.05

### Perioperative data

Surgery was successfully performed in 77 patients. No intraoperative complications were reported in both study groups. Mean operation time (defined as time from incision to skin closure) was longer for patients who underwent lumbopelvic fixation than for patients who were treated with ISF (165.0 ± 68.0 min. vs. 73.0 ± 46.0 min.; respectively, *p* < 0.001).

No significant differences were found between the two groups regarding fluoroscopy time (LPF group: 4.88 ± 6.13 min, ISF group: 6.33 ± 4.62 min; *p* = 0.28) and radiation exposure (LPF group: 2708.78 ± 3106.97 Gy*cm^2^, ISF group: 3136.64 ± 2066.76 Gy*cm^2^; *p* = 0.51) during surgery. Hardware removal was performed in five patients (11.6%) with ISF and in 2 cases (6.9%) treated with LPF.

### Revisions/complications

There was no intraoperative death and no patient deceased during hospital stay due to postoperative complications. The most common postoperative complications encountered in our series included hematoma or infection in the affected surgical region. Deep wound infections were related to lumbopelvic fixations compared to the ISF group (*n* = 4 versus *n* = 1; respectively, *p* = 0.008). Patients with hematoma or wound infection underwent local debridement and IV followed by oral antibiotic administration.

Postoperative CT revealed screw malpositioning after ISF in 5 patients with consecutive hardware change. There was no malpositioning of screws or rods in the LPF group.

In our study group, one patient experienced nonunion formation with consecutive hardware failure (LPF group: *n* = 1, ISF group: *n* = 0). Consecutive surgical revision consisted of rod replacement and autologous bone grafting. When comparing those who sustained complications to those who did not in the LPF or ISF group, there were no significant differences with respect to age or BMI.

### Outcome/weight bearing

Following biomechanical considerations, patients who underwent LPF were allowed to weight-bear as tolerated immediately, whereas patients with ISF were restricted from weight bearing on their lower extremities for 12 weeks (*p* < 0.001) depending on the injured pubic rami (left, right or both). In contrast, patients with ISF were significantly earlier discharged to home or to a skilled nursing facility compared to the LPF group (13.0 ± 7.7 days vs. 26.6 ± 17.9 days; respectively, *p* = 0.001).

## Discussion

Operative treatment of traumatic posterior pelvic ring disruption can be complex and technically demanding but residual posterior displacement is associated with poor functional outcomes [26]. Moreover, malreduction can lead to significant long-term disabilities due to pain, leg length discrepancy, nonunion formation and neurological compromise [27]. Therefore, the main objectives of operative treatment for unstable posterior pelvic ring fractures are fracture reduction and rigid fixation to facilitate early mobilization.

Biomechanical studies have confirmed that segmental lumbopelvic stabilization provides stable fixation of the posterior pelvic ring and allows early weight bearing [11, 28–30]. The procedure is demanding with numerous potential complications especially related to soft tissue involvement. High rates of fixation failure and local pain have been reported and restriction of the technique combined with routine hardware removal has been advocated [13]. On the other hand, high success rates can be achieved when it is performed systematically and in appropriately selected patients [6]. Prominence of iliac screw heads was a frequent problem in thin patients. In previous studies, up to 95% of the patients had painful and prominent implants. Therefore, routine hardware removal has been required [13, 31]

In contrast, Routt et al. [14] recommended closed reduction and percutaneous fixation (CRPF) for posterior pelvic ring disruption. The advantages to percutaneous ISF include avoidance of a lower lumbar spine fusion, decreased invasiveness with relatively low soft tissue trauma and a reasonable blood loss. A considerable disadvantage is the fact, that patients were still not able to support full weight bearing. In several studies, percutaneous ISF showed good function results and low rate of complications [32–34].

Our study can provide important data regarding surgical treatment of bilateral sacral fractures and may be useful in the clinical management of these injuries. A gold standard algorithm does not exist for the treatment of these patients. Thus, this comparison of the two surgical procedures may suggest important patients’ characteristics that aid in the decision-making process for one of the two surgeries.

In this study, comparisons were made between demographic, perioperative data and complications after LPF and ISF based on the data of patients with at least 6-month follow-up. Our patients, with a mean age of 71 years and being 74% female, were representative for the typical patients group with osteoporotic posterior ring fractures after low-energy trauma [35]. Regarding demographic data, we found significantly older ISF patients with a predominance of women compared to the LPF group. A German multicenter study showed similar results with a higher incidence of pelvic fractures for patients older than 65 years with more women than men [36]. The differences in demographics may suggest that surgeons tend to less invasive procedures in elderly patients. Therefore, additional prospective studies are warranted.

As expected, the operative time was significantly longer in those patients that underwent LPF. Kelly et al. [37] also compared ISF and LPF in case of U/H type sacral fractures. Those that were treated with LPF also had a significantly higher operative time, likely due to the concurrent sacral decompression at the time of surgery. Overall, their surgical times for LPF 326 min) and for ISF (89 min) were obviously higher compared to our results (LPF: 165.0 ± 68.0 min, ISF: 73.0 ± 46.0). Hopf et al. [38] examined ISF after osteoporotic posterior ring fractures of the pelvis in elderly patients and exhibited good clinical results with less intra- and postoperative complications. They regarded this type of surgery as a safe procedure. Therefore, older patients that require fixation of bilateral sacral fixation may be better suited for treatment with ISF if there is concern that the patient may not tolerate the increased operative time. Additional cement augmentation of iliosacral screws may increase stability in osteoporotic patients, but was not performed in our patient population [39]. On the other hand, despite a longer operative time, patients with an elevated risk of pneumonia or other pulmonary complication may benefit of immediate weight bearing with LPF. This is evidenced by the fact that pulmonary complications were common in patients with pelvic fractures [40].

Within the current literature, there are very few comparative data to inform the treating surgeon on the issue of weight bearing after pelvic fracture surgery [41]. In our institution, patients with ISF are usually restricted from bearing weight on their lower extremities for 12 weeks. Nork et al. [42] recommended similar restriction. Despite the strict restriction of mobilization of the ISF group, these patients were significantly earlier discharged to home or to a skilled nursing facility (13.0 ± 7.7 days) compared to the LPF group (26.6 ± 17.9 days). Therefore, the postoperative restriction of weight bearing was not decisive for a longer hospital stay. Sathiyakumar et al. [43] demonstrated a significant difference in the length of stay in patients undergoing open reduction and internal fixation (ORIF) versus percutaneous fixation of sacral injuries. Patients with ORIF required an extended hospital stay for approximately three additional days.

Based on our results for LPF or ISF, hardware displacement and infection were the two main reasons for complications. Avoidance of deep tissue exposure to the environment in percutaneous ISF is one main reason that is postulated to result in theoretically lower complication rates with this approach compared to open techniques [44].

Our study showed 2.1% infection rate in case of ISF. As already suspected, the infection rate after LPF was distinctly higher (13.8%). Compared with the current literature our infection rates are representative for this kind of treatment [45–48]. For example, Matta et. al showed 2.8% infections in internal fixation [49]. Bellabarba et al. [48] described infection rates up to 16% of patient after open LPF, which is close to our 13.8%.

We figured out a trend toward an increased screw malpositioning rate in the ISF group (*n* = 5) compared to the LPF group (*n* = 0, *p* > 0.07). Consequently, we had a screw malpositioning rate of 10.6%. Different studies showed displacement rates after ISF between 8 and 13% [50–53]. Screw malpositioning rate can result from poor visualization of relevant anatomy, unexpected anatomic variations, incomplete fluoroscopy and malreduction [54]. Postoperatively, the exact control of implant positions is unchangedly necessary because no implantation method of iliosacral or lumbopelvic screws can prevent malplacement of screws.

In our series, there was no significant difference in frequency of routine hardware removal in both study groups. Merely two patients who underwent LPF needed a second surgery to remove the instrumentation. We did not perform a routine hardware removal unless it is causing discomfort. In our opinion, the hardware removal in the pelvic ring should be strictly defined.

The weaknesses of this study relate to the small patient number due to the sparsity of the fracture. Furthermore, our study is retrospective and thus has the limitations inherent to retrospective analysis. The small number and large variances could skew final outcome measurements and conclusions. Several surgeons were involved in the study and the procedure performed was based on clinical preference and judgment.

The strength of this study is the number of patients included. To our knowledge this study is the largest consecutive single center series. The retrospective design of the study may also offer the advantage that all patients were treated for their benefit and not for comparing two different treatment philosophies.

## Conclusion

Posterior pelvic ring injuries with bilateral fractures of the sacrum benefit from LPF and ISF without an expected difference in fluoroscopic time, radiation exposure or routine hardware removal. However, treatment with ISF decreases overall operative time, length of hospitalization and postoperative wound infection, significantly. Older patients may be better suited for treatment with ISF if there is concern that the patient may not tolerate the increased operative time. On the other hand, patients with an elevated risk for pneumonia or other pulmonary complication may benefit of immediate weight bearing with LPF.

## Data Availability

The datasets used and/or analyzed during the current study are available from the corresponding author on reasonable request.

## Abbreviations

LPF: Umbopelvic fixation
ISF: Iliosacral screw fixation
SIJ: Sacroiliac joint
CPT: Current Procedural Terminology
CT: Computed tomography
BMI: Body mass index
CRPF: Closed reduction and percutaneous fixation
ORIF: Open reduction and internal fixation
